# Untargeted Metabolomic Signature of Mice with Ischemic Stroke

**DOI:** 10.3390/metabo16080544

**Published:** 2026-07-31

**Authors:** Wenqi Zhang, Xinyao Ju, Xueyun Xie, Shizhen Song, Tingting Zhang, Shuang Zhou

**Affiliations:** School of Acupuncture-Moxibustion and Tuina, Shanghai University of Traditional Chinese Medicine, Shanghai 201203, China; zhangwenqi012@163.com (W.Z.); jxyjuwendy@163.com (X.J.); xiexueyun@163.com (X.X.); sejins@163.com (S.S.); zhangtt1999@126.com (T.Z.)

**Keywords:** ischemic stroke, MCAO, metabolomics

## Abstract

**Background:** Ischemic stroke (IS) became the most common type of stroke in 2021, leading to an extreme health burden globally, and has the potential to further increase the burden in the young. However, current therapies for IS are limited, and its mechanism remains unclear. **Methods:** SPF C57BL/6J male mice were used to establish the middle cerebral artery occlusion (MCAO) model. The Longa’s Neurological Deficit Score and TTC staining served as evaluative metrics for the efficacy of the model. Untargeted metabolomics analysis was performed based on LC-MS technology, and PCA, PLS-DA, and KEGG analysis were used to explore the differential metabolites. **Results:** Compared to the Sham group, the neurological deficit score of the MCAO group was significantly higher at 2 h and 72 h after surgery (*p* < 0.001). There was no infarct volume in the Sham group, while the infarct volume ratio of the MCAO group significantly increased compared with that of the Sham group. In total, 1836 metabolites were identified overall, and they were clustered into 14 classifications, with “lipids and lipid-like molecules” accounting for the most (34.45%). Compared with the Sham group, 95 metabolites were up-regulated, and 107 metabolites were down-regulated in the MCAO group; 138 of 202 were identified with HMDB numbers. A further clustering and KEGG pathway analysis showed that these 138 metabolites may be involved in lipid metabolism, amino acid metabolism, and nucleotide metabolism. However, FDR was not used as extracting criteria to screen out the differential metabolites, which indicated that the outcomes should be considered for further validation. **Conclusions:** This discovery-stage untargeted metabolomics analysis suggests that ischemic stroke may be associated with alterations in lipid, amino acid, and nucleotide metabolism in the mouse brain, but these exploratory findings require validation in larger independent cohorts using targeted metabolomics.

## 1. Introduction

Stroke is a common noncommunicable neurological disease and has led to an extreme health burden globally; it was the third most common cause of death and the fourth of disability-adjusted life-years (DALYs) in 2021, and stroke mortality will increase by 50% from 2020 to 2050 according to the WSO/Lancet Neurology Commission on Stroke [1,2]. Ischemic stroke (IS) is the most common type worldwide, accounting for 65.3% of all kinds of strokes in 2021, and its burden may be increasing further in the young [3,4,5]. A sudden decrease in O2 and nutrients in the brain due to vascular obstruction caused by blood clots or narrowing of blood vessels is the main cause of IS, which could lead to permanent neurological damage. However, the effects of current medical therapies are limited, and the precise mechanism of brain ischemia remains unclear.

Studies showed that neural cell death, oxidative stress, and inflammation could be the main three reasons for ischemic stroke [6,7]. Brain ischemia and infarcts directly cause the damage and loss of neurons, leading to different types of cell death. Researchers have been focusing on neuron-cell protection and regeneration, as well as related biomarkers and pathways based on this basic mechanism [8,9]. In addition, extra reactive oxygen species (ROS) caused by ischemia could exacerbate neuronal damage and cell death. Scavenging extra ROS in the brain could be effective in neural and microvessel protection [10,11]. Inflammation is another crucial risk factor of cerebral ischemic injury. Inflammatory factors induced by ischemia and hypoxia, such as INF-α and IL-6, could directly cause neuronal dysfunction and cellular damage [12,13].

Metabolomics studies have revealed distinct metabolic alterations, including amino acid metabolism, lipid metabolism, and glucose metabolism in the ischemic and hypoxic environment [14,15]. As an essential metabolite, amino acids are involved in various physiological and pathological processes of the central nervous system. In brain ischemia, excessive glutamate, homocysteine, and phenylalanine are crucial factors of neuron and vascular infarcts, which could lead to oxidative stress and cell death [16,17,18]. Lipids are structural components of cell membranes and serve as signalling molecules. Unsaturated fatty acids, including docosahexaenoic acid (DHA) and eicosatetraenoic acid (EPA), have been verified beneficial in brain ischemia reduction, while hydrolyzed Phosphatidylcholine (PC) can lead to inflammation, lipid peroxidation, and cell damage [19,20]. The normal function of neurons and astrocytes depends on enough energy from glucose metabolism. IS triggers disruption of oxygen utilization and increased glycolytic flux, leading to ROS elevation and lactic acid buildup, which is deleterious to the survival of cells in the ischemic penumbra [21,22].

As an emerging systematic science, metabolomics employs analytical platforms such as mass spectrometry (MS), Nuclear Magnetic Resonance (NMR), and Ultra Performance Liquid Chromatography (UPLC) to systematically identify and quantify the small-molecule metabolites (<1500 Da) within specific samples, establishing an association between metabolic signature and disease phenotypes [23,24]. Compared with proteomics and transcriptomics, metabolomics has more strengths in uncovering biomarkers of IS because it can detect small-molecule metabolites that cross the blood–brain barrier and provide a relatively accurate representation of the chemical intermediates or endpoints [25,26]. Moreover, untargeted metabolomics enables wider detection of metabolites in the brain, providing more possibilities in realizing the complex mechanisms of IS [27]. Over the past decades, metabolomics has shown great promise in mechanism exploration, and a range of IS-related biomarkers and metabolic pathways have been discovered [15,28,29]. Therefore, metabolomics reflects and solves the complexity of IS research to some extent. However, metabolomics data related to IS are still limited, and few previous studies have explored the metabolic signature of the brain ischemia area.

This study used permanent middle cerebral artery occlusion (MCAO) and aims to draw the metabolite signature of the brain ischemia area based on the LC-MS untargeted metabolomics method, providing discovery-stage experimental evidence for the mechanism of brain ischemia infarcts.

## 2. Materials and Methods

### 2.1. Experiment Animals and Groups

Male SPF C57BL/6J mice weighing 20–25 g at 6–8 weeks were purchased from Zhejiang Vital River Laboratory Animal Technology Co., Ltd. (Shanghai, China), and raised in the Animal Experiment Center of Shanghai University of Traditional Chinese Medicine with the Animal Ethics Committee (PZSHUTCM2411120001, approved on 5 December 2025). The housing environment maintained controlled temperature (20 ± 2 °C), humidity (50–70%), lighting (12 h light/dark cycle), and all the mice were allowed to acclimate in the individually ventilated cages (IVC) systems for 7 days before the formal experiment with free access to food and water, as well as unrestricted movement within the cage environment. The 24 mice were randomly divided into two groups using a random-number method (Sham and MCAO), with 12 mice in each group. Neurological deficits score was evaluated in all animals. At 72 h after surgery, 6 mice from each group were used for TTC staining and infarct volume assessment, and the remaining mice from each group were used for metabolomics analysis. Neurological deficit scoring and TTC infarct assessment were performed by investigators blinded to group allocation. For metabolomics analysis, sample labels were coded before LC-MS analysis and data processing to reduce operator bias.

### 2.2. MCAO Establishment

After 12 h of fasting, mice were anesthetized intraperitoneally with 1% sodium pentobarbital (50 mg/kg, Sigma-Aldrich, St. Louis, MO, USA). The mouse MCAO model was established following the method reported by Hata: (1) we made a median carotid incision, separated the subcutaneous tissues, exposed the right carotid sheath, and separated the carotid artery and vagus nerve bluntly; (2) we then freed the common carotid artery (CCA) to the bifurcation, ligated the external carotid artery and CCA, and clipped the internal carotid artery with vascular clips; (3) we cut the CCA from the proximal end with microscopic ophthalmic scissors, and a nylon monofilament (Cinontech, Beijing, China) with a rounded tip was inserted into the ICA and stopped when it was met with slight resistance to occlude the entrance of the middle cerebral artery; (4) the CCA was gently ligated just superior to the incision site to firmly secure the thread embolus with sutures, followed by the routine closure of the wound. The temperature of the heating pad was maintained at 30.0 ± 0.5 °C during the whole operation. After the surgery, the mice were moved to the cages, and care was taken to maintain their body temperature until awake. In the Sham group, the vessels were separated with no nylon monofilament insertion in operation, and the remaining steps were the same as above.

### 2.3. Neurological Deficits Score

Longa’s Neurological Deficit Score was used to assess 2 h and 72 h after the surgery, and scores of 2 to 3 were considered valid models for inclusion: (1) 0 points: no obvious defect. (2) 1 point: flexion of the trunk on the ischemic contralateral side after tail lifting, or inability to straighten the ischemic contralateral forelimb recovery. (3) 2 points: spontaneous turning to the ischemic contralateral side while walking on a 1-point basis, but no abnormal position at rest. (4) 3 points: tilting of the body to the ischemic contralateral side or tipping over while resting. (5) 4 points: the absence of any spontaneous movement, and 5 points for death before the assessment.

### 2.4. The 2,3,5-Triphenyltetrazolium Chloride (TTC) Staining

Concerning Benedek, the whole brain tissue was frozen at −20 °C for 10 min, and then the tissue was evenly cut with a total of 5 slices from the coronal plane of the optic cross (forward 1 slice and backward 4 slices of 2 mm thick slices), which were put into 1% TTC (Sigma-Aldrich, St. Louis, MO, USA) solution, incubated at a constant temperature of 37 °C and avoiding light for 30 min, and turned over every 15 min. After that, the slices were transferred to 4% paraformaldehyde to fix for 24 h at 4 °C to take tissue photos. Image J 1.54 was used to calculate the size of the infarct area slices. The volume of the infarct area in brain slices was calculated according to the formula: Infarct volume = (contralateral area − ipsilateral non-infarct area) × thickness of brain slices. The volume of 5 slices acted as the total volume. The infarct volume ratio was calculated according to the formula: Infarct Volume Ratio = [Infarct volume/ (2 × ipsilateral non-infarct area × thickness of brain slices)] × 100%.

### 2.5. Metabolomic Sample Preparation

The sample preparation was performed as follows: (1) At 72 h after surgical procedures, mice were anesthetized and perfused with pre-chilled PBS solution to remove blood from tissues. (2) Rapid decapitation was performed, and craniotomy was performed, and then the meninges were dissected to expose the brain on ice. (3) We removed the right cerebral cortex and moved it into a labelled cryopreservation tube. (4) We froze the tissue in liquid nitrogen for 15 min and then stored it in a −80 °C freezer for cryopreservation. The detailed procedures for metabolite extraction were as follows: (1) Weigh 20 mg of liquid nitrogen-ground tissue samples into EP tubes and add 100 μL of 80% aqueous methanol solution to each tube. (2) After vortex oscillation, the samples were incubated on ice for 5 min, followed by centrifugation at 15,000× *g* and 4 °C for 20 min. (3) Take an appropriate volume of the supernatant and dilute it with mass spectrometry-grade water to a final methanol concentration of 53%. (4) Centrifuge the diluted solution at 15,000× *g* and 4 °C for 20 min, collect the supernatant, and perform untargeted metabolomics analysis via LC-MS.

To ensure experimental quality control, quality control (QC) samples were prepared concurrently with experimental samples. These QC samples consisted of equal-volume mixtures of experimental samples, serving to balance the chromatography–mass spectrometry system and monitor instrument status.

### 2.6. Metabolomic Analysis

#### 2.6.1. Chromatographic Conditions

Chromatographic column: Hypersil Gold column (C18). Column temperature: 40 °C. Flow rate: 0.2 mL/min. Mobile phase A: 0.1% formic acid. Mobile phase B: Methanol (Table 1).

#### 2.6.2. Mass Spectrometry Conditions

Mass spectrometric detection was performed over an *m*/*z* range of 100–1500 in both positive- and negative-ion modes. The electrospray ionization source parameters were set as follows: spray voltage, 3.5 kV; sheath gas flow rate, 35 arbitrary units; auxiliary gas flow rate, 10 arbitrary units; capillary temperature, 320 °C; S-lens RF level, 60; and auxiliary gas heater temperature, 350 °C. Tandem mass spectrometry data were acquired using a data-dependent acquisition mode.

#### 2.6.3. Data Preprocessing and Metabolite Identification

The raw LC-MS/MS data files were imported into Compound Discoverer 3.3 for data preprocessing and metabolite annotation. Metabolic features were initially detected and aligned according to retention time and mass-to-charge ratio (*m*/*z*). Peak-area correction was performed using the first quality-control sample to improve intersample comparability.

Peak extraction was conducted using a precursor mass tolerance of 5 ppm and a signal-intensity tolerance of 30%, together with predefined minimum-intensity and adduct-ion criteria. Target ions were subsequently integrated, and their relative peak areas were obtained. Molecular formulas were predicted based on precursor ions, isotopic patterns, and fragment ions. The acquired MS/MS spectra were then matched against the mzCloud, mzVault, and MassList databases. Background features were removed using blank samples. The original peak areas were normalized according to the following formula: Normalized relative peak area = original peak area of a metabolite in a sample/(sum of the peak areas of all metabolites in that sample/sum of the peak areas of all metabolites in the first QC sample).

Metabolic features with coefficients of variation greater than 30% across the QC samples were excluded. The final dataset contained putatively annotated metabolites and their normalized relative peak areas. Metabolite annotation confidence was reported according to the Metabolomics Standards Initiative criteria. Compounds supported by accurate mass, predicted molecular formula, and MS/MS spectral-library matching, but not confirmed using authentic reference standards analyzed under identical experimental conditions, were classified as MSI Level 2 putatively annotated compounds.

#### 2.6.4. Statistical Analysis

The identified metabolites were annotated using the KEGG database (https://www.genome.jp/kegg/pathway.html, accessed on 3 June 2026), HMDB database (https://hmdb.ca/metabolites, accessed on 3 June 2026), and LIPID MAPS database (http://www.lipidmaps.org/, accessed on 3 June 2026). For multivariate statistical analysis, metabolomic data processing software metaX version 1.4.16 [30] was used to transform data before performing principal component analysis (PCA) and partial least squares discriminant analysis (PLS-DA), thereby obtaining the Variable Importance in the Projection (VIP) values for each metabolite. A permutation (*n* = 200) test was performed to assess the risk of overfitting for the PLS-DA model, and the model evaluation parameters (R2, Q2) were obtained after 7-cross-validation. The closer the values of R2 and Q2 are to 1, the more stable and reliable the model is. When the R2 value is greater than Q2, and the intercept of Q2 regression with the Y-axis is less than 0, it can be concluded that the model is not overfitting [31]. The fold change (FC) value, representing the relative change in metabolite levels between groups, was also computed. Metabolites meeting the criteria of VIP > 1.0, FC > 1.2 or FC < 0.833 with *p* < 0.05 were selected as differentially expressed metabolites in this study and false discovery rate (FDR) correction was applied in the Supplementary Materials. Correlation analysis (Pearson correlation coefficient) between differential metabolites was conducted via the *cor*() function in R, and statistical significance was verified by the *cor.mtest*() function in R, with *p* < 0.05 considered statistically significant. For volcano plots, outcomes were plotted with the R package *ggplot2*, and differential metabolites were screened by integrating three parameters of metabolites: VIP value, log_2_(FC) and log_10_(*p*-value). Clustered heatmaps were generated using the R package *Pheatmap*, with metabolite data normalized by z-score. The relevant metabolic pathways were identified using MetaboAnalyst (https://www.metaboanalyst.ca/, accessed on 3 June 2026). A metabolic pathway was considered enriched when x/n > y/n and the *p*-value of the metabolic pathway was <0.05.

## 3. Results

### 3.1. Brain Ischemia Infarction

**Figure 1:**
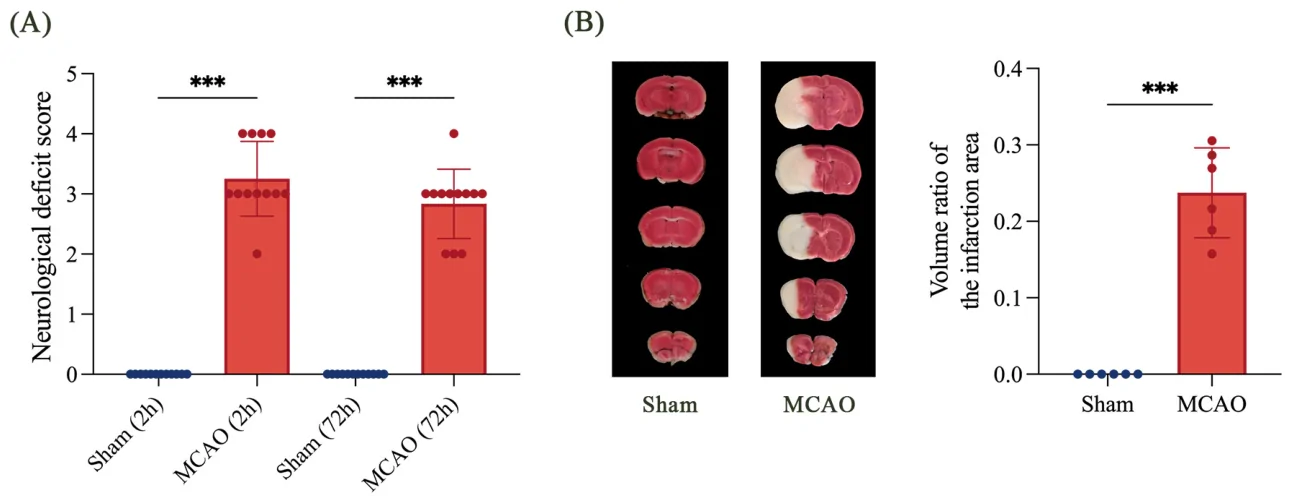
Brain ischemia infarction. (**A**) Neurological deficits scores of mice (*n* = 12). Compared to the Sham (2 h) group, *** *p* < 0.001. (**B**) The TTC staining and the volume ratio of ischemic brain in mice. Compared to the Sham group, *** *p* < 0.001.

The median and interquartile range of the neurological deficit scores in the Sham group were 0 at both 2 h and 3 d. The scores showed that the MCAO group was higher than the Sham group at 2 h postoperatively (*p* < 0.001, Figure 1A). To better evaluate the condition of MCAO, the volume of cerebral infarction and the angiogenesis were calculated. There was no infarct volume in the Sham group, while the infarct volume ratio of the MCAO group significantly increased compared with that of the Sham group (*p* < 0.001), which indicated that MCAO model establishment leads to a severe brain infarction (Figure 1B).

### 3.2. Comprehensive Metabolite Analysis

**Figure 2:**
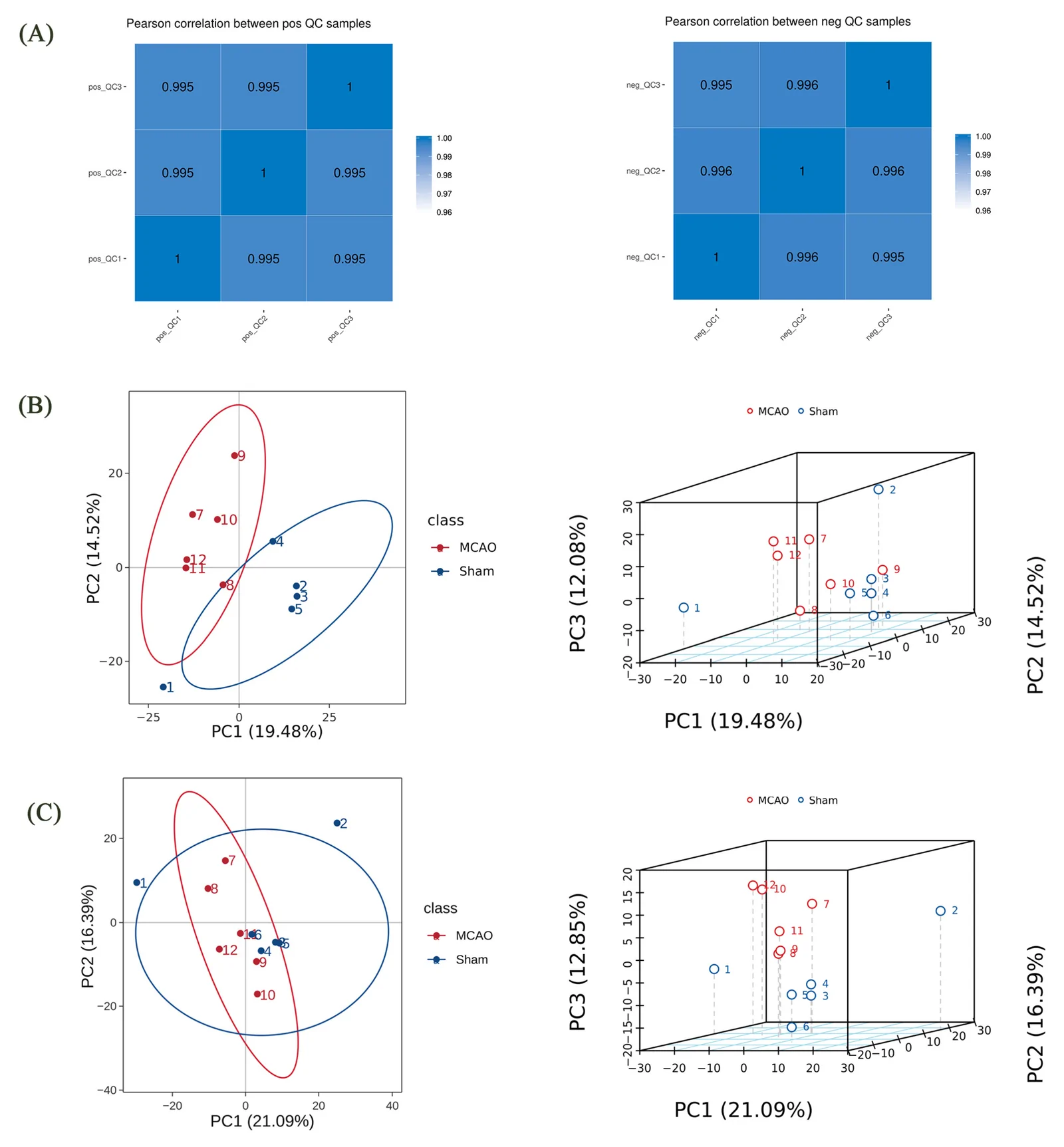
Pearson correlation and PCA. (**A**) Pearson correlation of QC samples; the left is positive mode, and the right is negative mode. (**B**) PCA of the Sham and MCAO groups in positive mode. (**C**) PCA of the Sham and MCAO groups in negative mode. **Notes:** QC: quality control, PC: principal component.

To determine the metabolic characteristics of the brain, qualitative and quantitative analyses were performed initially, before which the Pearson correlation coefficients between QC and experimental samples based on the relative quantitative values of metabolites were calculated, and a higher correlation among QC samples (the closer |r| is to 1) indicated a better stability of the testing process and higher data quality. Outcomes of the Pearson correlation showed that all |r| were above 0.995, which indicated a stable detection process with high-quality data output, and the metabolite data could be used for further analysis (Figure 2A).

After normalizing the samples, PCA was employed to determine the metabolic differences between the Sham and MCAO groups and the degree of variation within each group. The results showed that samples in each group clustered tightly, suggesting reliable sample stability. Although in positive mode there was a partial overlap of metabolic profiles in the brain tissue with and without MCAO operation, a significant difference still appeared (Figure 2B). Additionally, metabolic profiles that showed high overlap in negative mode also exhibited clear separation when it was expanded to three principal components, indicating that metabolic characteristics of the brain tissue changed significantly after MCAO operation (Figure 2C).

**Figure 3:**
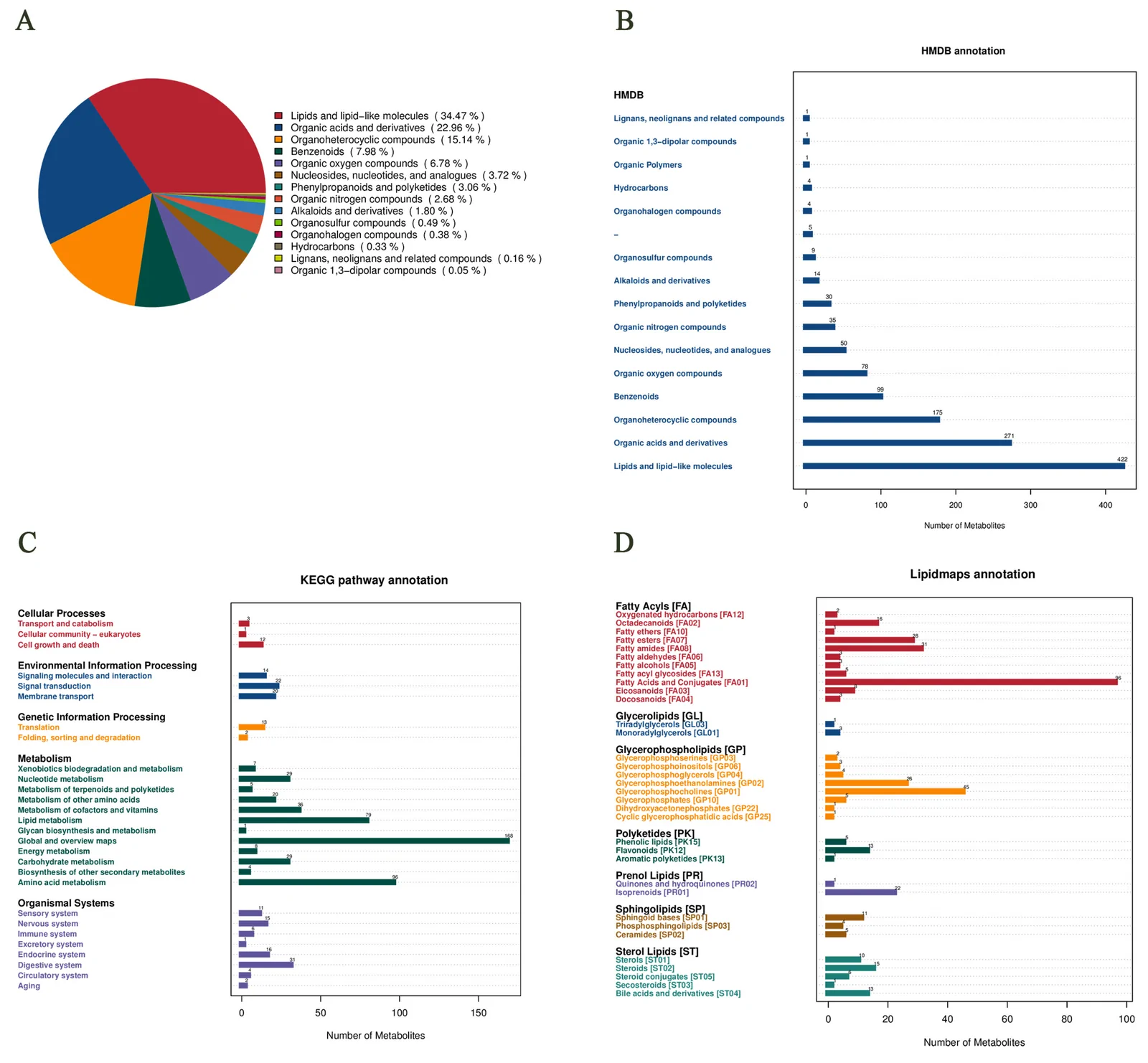
Overall profile of the identified metabolites. (**A**) Pie chart of the class distribution of the 1836 identified metabolites. (**B**) HMDB annotation of metabolites. (**C**) KEGG annotation of metabolites. (**D**) Lipidmaps annotation of identified metabolites.

**Figure 4:**
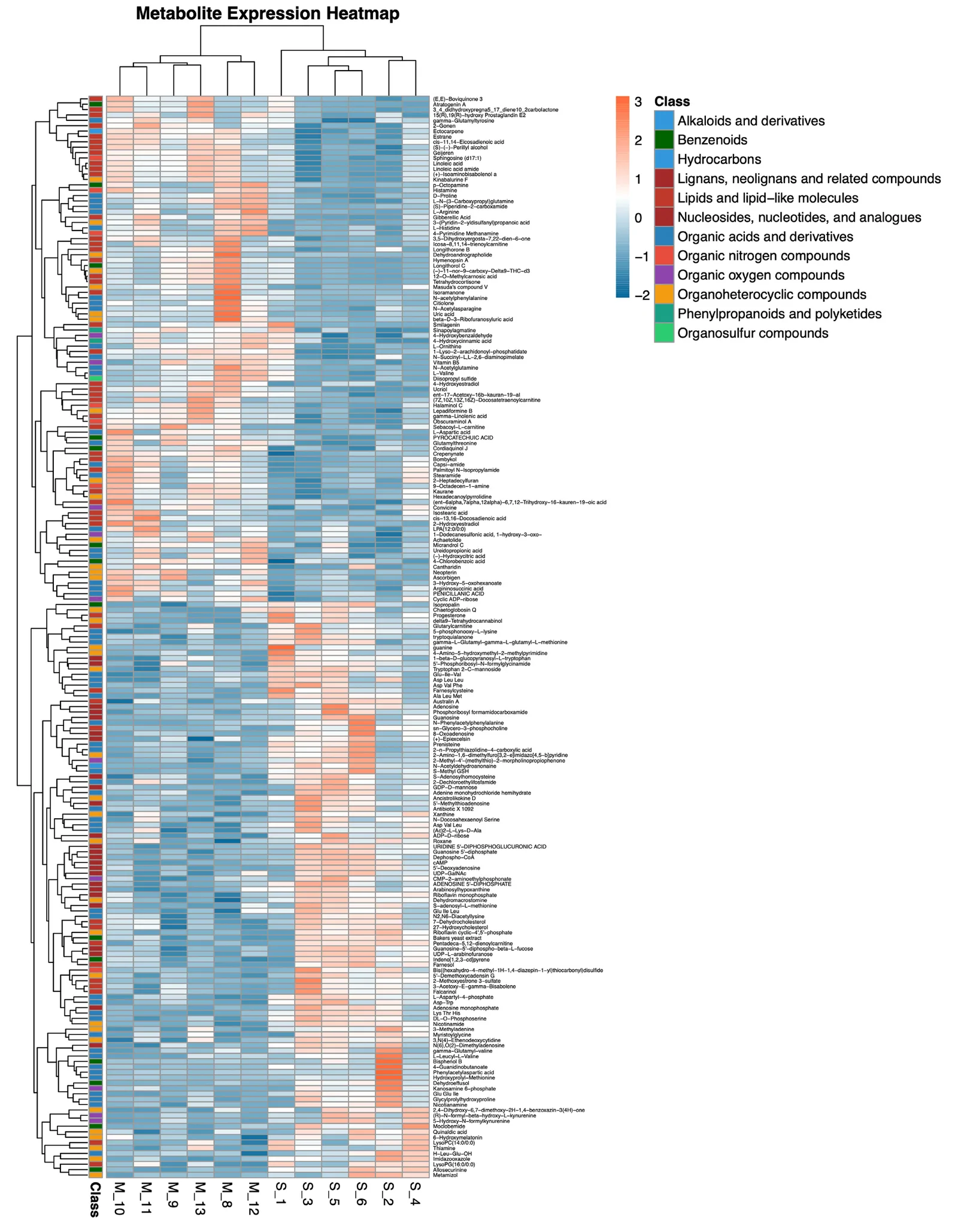
Heatmap of 202 identified differently expressed metabolites in the two groups (*p* < 0.05). The colours changing from red to blue indicate the expression level variations in metabolites, the red plots indicate the up-regulated metabolites, and the blue plots indicate the down-regulated metabolites.

By comparing the metabolite data against a standard metabolite database, 1836 metabolites were identified, and they could primarily be clustered into “lipids and lipid analogues” (34.47%), “organic acids and derivatives” (22.96%), “organ heterocyclic compounds” (15.14%), “benzenoids” (7.98%), “organic oxygen compounds” (6.78%), “nucleosides, nucleotides, and analogues” (3.72%), etc. (Figure 3A). Based on the HMDB and KEGG pathway database, we conducted an overview classification of all the observed metabolites to identify the essential metabolic characteristics of the brain. As a result, “lipids and lipid-like molecules” accounted for the most in HMDB (422 annotations, Figure 3B), and the metabolites related to kinds of life processes, including cellular growth and death, signal transduction, and amino acid metabolism (Figure 3C). In addition, we performed lipids analysis based on the LIPID MAPS database as “lipids and lipid-like molecules” were the most identified metabolites, and seven categories of Lipidmaps annotation including Fatty Acyls (FA), Glycerolipids (GL), Glycerophospholipids (GP), Polyketides (PK), Prenol Lipids (PR), Sphingolipids (SP) and Sterol Lipids (ST) were recorded in the overall metabolites (Figure 3D). Then VIP values, FC value, and *p*-value based on the T-test were calculated to identify the differential metabolites in ischemic brain between the two groups, and 202 metabolites were screened out in total (VIP > 1.0, FC > 1.2 or FC < 0.833 with *p* < 0.05). To visually observe the differences in metabolites, a heatmap was performed, and it revealed similarity in distribution and types of metabolites across the Sham and MCAO group, while significant differences still existed in specific metabolites after the MCAO operation (Figure 4).

### 3.3. Metabolite Changes After Stroke

**Figure 5:**
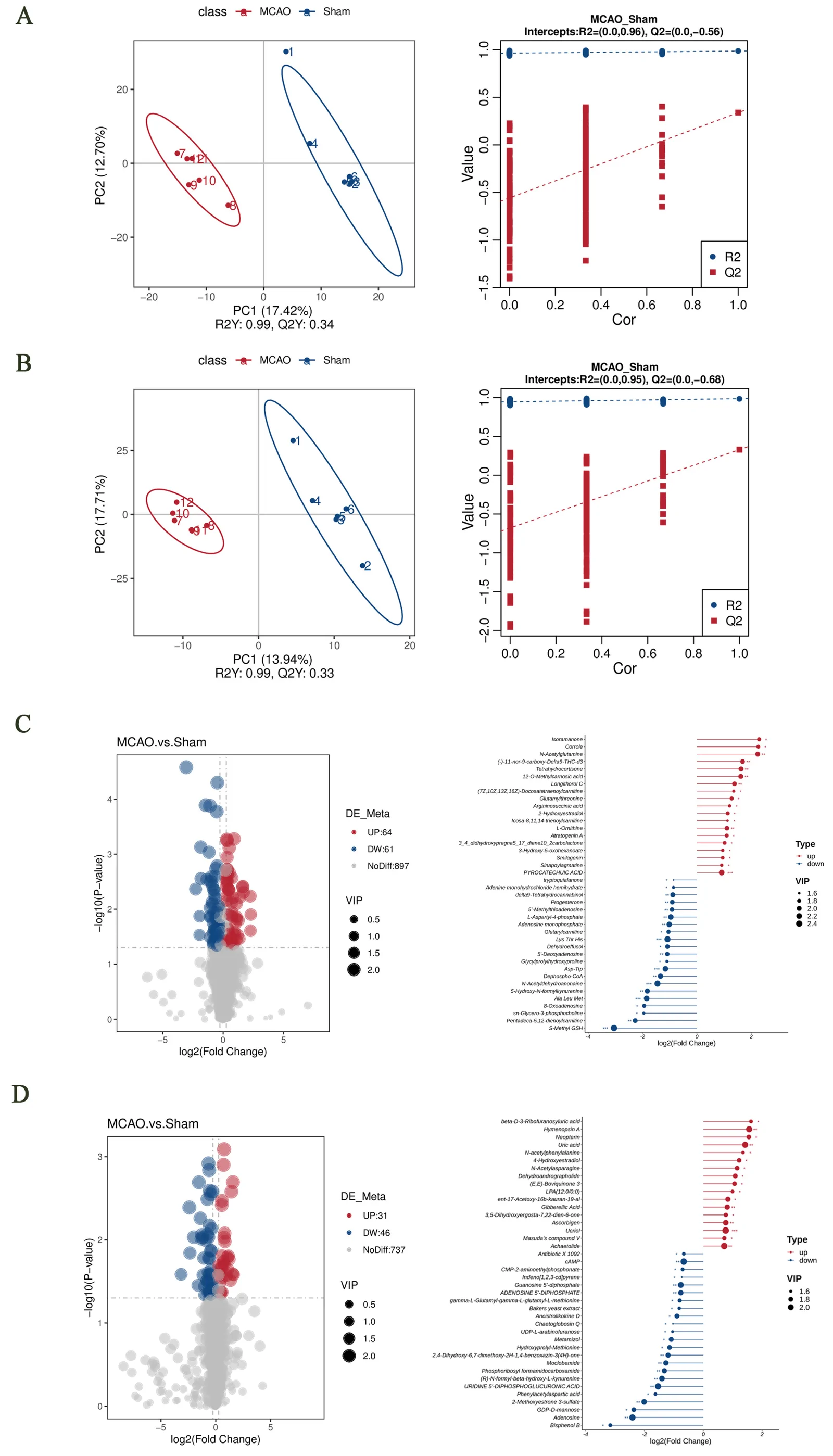
Differences in metabolites in the brain before and after MCAO. (A) PLS-DA score plot and for the Sham and MCAO groups in positive mode; PLS-DA permutation testing graph (*n* = 200) in positive mode. (B) PLS-DA score plot and for the Sham and MCAO group in negative mode; PLS-DA permutation testing graph (*n* = 200) in negative mode. (C) Volcano and scatter plot of the differential metabolites in the Sham and MCAO groups in positive mode. (D) Volcano and scatter plot of the differential metabolites in the Sham and MCAO groups in negative mode. * *p* < 0.05, ** *p* < 0.01, *** *p* < 0.001.

To more precisely identify the metabolite differences between the Sham and MCAO groups, PLS-DA analysis and permutation test (*n* = 200) were performed, which constructed a supervised statistical relationship model between the two groups using PLS regression analysis. Obviously, samples in each group clustered tightly, and a clear separation trend was observed in the PLS-DA model (in positive mode, R2Y = 0.99, Q2Y = 0.34, intercepts: R2 = 0.96, Q2 = −0.56, Figure 5A; in negative mode, R2Y = 0.99, Q2Y = 0.33, intercepts: R2 = 0.95, Q2 = −0.68, Figure 5B). However, given the moderate Q^2^ values and relatively small sample size, the model should be interpreted as exploratory, and the possibility of overfitting cannot be completely excluded despite the permutation test results. Volcano and scatter plots showed that compared with the Sham group, 95 metabolites were up-regulated, and 107 metabolites were down-regulated (in positive mode, 64 metabolites were up-regulated, VIP > 1, FC > 1.2, *p* < 0.05, and 61 were down-regulated, VIP > 1, FC < 0.8, *p* < 0.05, Figure 5C; in negative mode, 31 metabolites were up-regulated, VIP > 1, FC > 1.2, *p* < 0.05, and 46 were down-regulated, VIP > 1, FC < 0.8, *p* < 0.05, Figure 5D) in the MCAO group. Then we excluded metabolites without an HMDB ID to perform analysis precisely, and found that 65 metabolites were upregulated, and 73 metabolites were downregulated. As shown in Table 1, the up-regulated metabolites primarily were lipids and lipid-like molecules (22/65, Table 2), organic acids and derivatives (20/65, Table 2), while nucleosides, nucleotides, and analogues (20/73, Table 3), and organic acids and derivatives (17/73, Table 3) accounted for more in the down-regulated metabolites. All these identified differential metabolites indicated that ischemic stroke changed the signature of the brain, and lipids, nucleosides, and organic acids could play a role in ischemic stroke.

**Table 2:** The up-regulated metabolites with HMDB ID.

Name	Class	Ion Mode	Formula	RT (min)	*m*/*z*	Adduct	HMDB_ID	log_2_FC	*p*-Value	VIP
PYROCATECHUIC ACID	Benzenoids	P	C_7_H_6_O_4_	1.082	192.990	[M + K]+	HMDB0000397	0.911	0.001	2.101
4-Chlorobenzoic acid	Benzenoids	N	C_7_H_5_ClO_2_	0.69	154.990	[M − H]−	HMDB0246393	0.268	0.026	1.841
*p*-Octopamine	Benzenoids	P	C_8_H_11_NO_2_	2.084	136.076	[M + H-H_2_O]+	HMDB0004825	0.853	0.043	1.545
Ucriol	Lipids and lipid-like molecules	N	C_20_H_32_O_3_	8.723	319.227	[M − H]−	HMDB0036705	0.761	0.001	2.332
Estrane	Lipids and lipid-like molecules	P	C_18_H_3_0	9.64	247.242	[M + H]+	HMDB0251974	0.322	0.001	2.058
1-Lyso-2-arachidonoyl-phosphatidate	Lipids and lipid-like molecules	N	C_23_H_39_O_7_P	8.907	457.236	[M − H]−	HMDB0012496	0.574	0.016	2.018
Gibberellic Acid	Lipids and lipid-like molecules	N	C_19_H_22_O_6_	2.395	345.133	[M − H]−	HMDB0003559	0.815	0.008	1.999
Linoleic acid amide	Lipids and lipid-like molecules	P	C_18_H_33_NO	9.137	280.263	[M + H]+	HMDB0062656	0.320	0.002	1.986
ent-17-Acetoxy-16b-kauran-19-al	Lipids and lipid-like molecules	N	C_22_H_34_O_3_	8.879	345.243	[M − H]−	HMDB0036722	0.837	0.010	1.982
Linoleic acid	Lipids and lipid-like molecules	P	C_18_H_32_O_2_	9.137	263.237	[M + H-H_2_O]+	HMDB0000673	0.338	0.002	1.972
gamma-Linolenic acid	Lipids and lipid-like molecules	P	C_18_H_30_O_2_	8.716	261.221	[M + H-H_2_O]+	HMDB0003073	0.310	0.003	1.942
Tetrahydrocortisone	Lipids and lipid-like molecules	P	C_21_H_32_O_5_	6.255	347.221	[M + H-H_2_O]+	HMDB0000903	1.624	0.004	1.897
(E,E)-Boviquinone 3	Lipids and lipid-like molecules	N	C_21_H_28_O_4_	5.999	343.191	[M − H]−	HMDB0030743	1.065	0.016	1.892
cis-11,14-Eicosadienoic acid	Lipids and lipid-like molecules	P	C_20_H_36_O_2_	9.922	291.268	[M + H-H_2_O]+	HMDB0005060	0.446	0.004	1.885
4-Hydroxyestradiol	Lipids and lipid-like molecules	N	C_18_H_24_O_3_	6.952	287.165	[M − H]−	HMDB0005896	1.219	0.017	1.872
3,5-Dihydroxyergosta-7,22-dien-6-one	Lipids and lipid-like molecules	N	C_28_H_44_O_3_	11.085	427.321	[M − H]−	HMDB0031953	0.769	0.020	1.837
2-Gonen	Lipids and lipid-like molecules	N	C_17_H_2_6	9.587	229.195	[M − H]−	HMDB0245136	0.288	0.027	1.775
(ent-6alpha,7alpha,12alpha)-6,7,12-Trihydroxy-16-kauren-19-oic acid	Lipids and lipid-like molecules	N	C_20_H_30_O_5_	6.994	349.201	[M − H]−	HMDB0036701	0.506	0.026	1.765
Isostearic acid	Lipids and lipid-like molecules	N	C_18_H_36_O_2_	10.899	283.264	[M − H]−	HMDB0031066	0.591	0.028	1.754
2-Hydroxyestradiol	Lipids and lipid-like molecules	P	C_18_H_24_O_3_	6.964	311.162	[M + Na]+	HMDB0000338	1.134	0.015	1.700
Smilagenin	Lipids and lipid-like molecules	P	C_27_H_44_O_3_	8.821	417.336	[M + H]+	HMDB0030048	0.950	0.026	1.651
Sebacoyl-L-carnitine	Lipids and lipid-like molecules	P	C_17_H_31_NO_6_	8.719	346.222	[M + H]+	HMDB0240726	0.526	0.028	1.590
(7Z,10Z,13Z,16Z)-Docosatetraenoylcarnitine	Lipids and lipid-like molecules	P	C_29_H_49_NO_4_	8.523	476.373	[M + H]+	HMDB0240758	1.359	0.034	1.571
Icosa-8,11,14-trienoylcarnitine	Lipids and lipid-like molecules	P	C_27_H_47_NO_4_	8.258	450.357	[M + H]+	HMDB0241578	1.125	0.036	1.527
cis-13,16-Docosadienoic acid	Lipids and lipid-like molecules	P	C_22_H_40_O_2_	10.642	319.299	[M + H-H_2_O]+	HMDB0062219	0.722	0.044	1.498
L-N-(3-Carboxypropyl)glutamine	Organic acids and derivatives	N	C_9_H_16_N_2_O_5_	1.934	231.098	[M − H]−	HMDB0029393	0.559	0.003	2.146
D-Proline	Organic acids and derivatives	P	C_5_H_9_NO_2_	1.398	116.071	[M + H]+	HMDB0003411	0.505	0.001	2.085
N-Acetylglutamine	Organic acids and derivatives	P	C_7_H_12_N_2_O_4_	1.427	189.087	[M + H]+	HMDB0006029	2.239	0.006	1.899
N-Acetylasparagine	Organic acids and derivatives	N	C_6_H_10_N_2_O_4_	1.377	173.056	[M − H]−	HMDB0006028	1.152	0.016	1.891
Citiolone	Organic acids and derivatives	P	C_6_H_9_NO_2_S	1.469	160.043	[M + H]+	HMDB0250301	0.742	0.012	1.845
L-Ornithine	Organic acids and derivatives	P	C_5_H_12_N_2_O_2_	1.183	133.097	[M + H]+	HMDB0000214	1.105	0.008	1.819
(-)-Hydroxycitric acid	Organic acids and derivatives	N	C_6_H_8_O_8_	1.249	189.004	[M-H-H_2_O]-	HMDB0031159	0.572	0.021	1.814
LPA(12:0/0:0)	Organic acids and derivatives	N	C_15_H_31_O_7_P	6.369	353.173	[M − H]−	HMDB0062319	0.993	0.027	1.774
N-acetylphenylalanine	Organic acids and derivatives	N	C_11_H_13_NO_3_	5.587	206.082	[M − H]−	HMDB0000512	1.351	0.031	1.741
Glutamylthreonine	Organic acids and derivatives	P	C_9_H_16_N_2_O_6_	1.412	249.108	[M + H]+	HMDB0028829	1.280	0.016	1.713
Capsi-amide	Organic acids and derivatives	P	C_17_H_35_NO	9.835	270.279	[M + H]+	HMDB0040940	0.590	0.013	1.712
L-Histidine	Organic acids and derivatives	P	C_6_H_9_N_3_O_2_	1.27	156.077	[M + H]+	HMDB0000177	0.372	0.023	1.660
Argininosuccinic acid	Organic acids and derivatives	P	C_10_H_18_N_4_O_6_	1.327	291.129	[M + H]+	HMDB0000052	1.203	0.029	1.608
Ureidopropionic acid	Organic acids and derivatives	P	C_4_H_8_N_2_O_3_	1.27	155.043	[M + Na]+	HMDB0000026	0.439	0.023	1.606
PENICILLANIC ACID	Organic acids and derivatives	P	C_8_H_11_NO_3_S	5.452	202.054	[M + H]+	HMDB0256227	0.571	0.035	1.591
L-Aspartic acid	Organic acids and derivatives	P	C_4_H_7_NO_4_	1.455	134.045	[M + H]+	HMDB0000191	0.287	0.037	1.566
N-Succinyl-L,L-2,6-diaminopimelate	Organic acids and derivatives	P	C_11_H_18_N_2_O_7_	1.441	291.118	[M + H]+	HMDB0012267	0.737	0.041	1.560
L-Arginine	Organic acids and derivatives	P	C_6_H_14_N_4_O_2_	1.284	175.119	[M + H]+	HMDB0000517	0.319	0.041	1.555
gamma-Glutamyltyrosine	Organic acids and derivatives	P	C_14_H_18_N_2_O_6_	4.814	311.124	[M + H]+	HMDB0011741	0.562	0.041	1.530
L-Valine	Organic acids and derivatives	P	C_5_H_11_NO_2_	1.455	118.086	[M + H]+	HMDB0000883	0.427	0.036	1.523
Stearamide	Organic acids and derivatives	P	C_18_H_37_NO	10.238	284.294	[M + H]+	HMDB0034146	0.506	0.038	1.494
9-Octadecen-1-amine	Organic nitrogen compounds	P	C_18_H_37_N	10.402	268.300	[M + H]+	HMDB0244390	0.830	0.006	1.840
Histamine	Organic nitrogen compounds	P	C_5_H_9_N_3_	1.27	112.087	[M + H]+	HMDB0000870	0.727	0.008	1.818
Convicine	Organic oxygen compounds	N	C_10_H_15_N_3_O_8_	1.348	304.078	[M − H]−	HMDB0250445	0.538	0.021	1.855
Vitamin B5	Organic oxygen compounds	N	C_9_H_17_NO_5_	4.948	218.103	[M − H]−	HMDB0000210	0.367	0.033	1.727
1-Dodecanesulfonic acid, 1-hydroxy-3-oxo-	Organic oxygen compounds	N	C_12_H_24_O_5_S	6.325	279.127	[M − H]−	HMDB0243866	0.599	0.043	1.668
Cyclic ADP-ribose	Organic oxygen compounds	N	C_15_H_21_N_5_O_13_P_2_	1.378	540.053	[M − H]−	HMDB0249529	0.468	0.045	1.650
4-Hydroxybenzaldehyde	Organic oxygen compounds	P	C_7_H_6_O_2_	2.082	123.044	[M + H]+	HMDB0011718	0.494	0.045	1.548
Uric acid	Organoheterocyclic compounds	N	C_5_H_4_N_4_O_3_	1.819	167.020	[M − H]−	HMDB0000289	1.425	0.003	2.193
Ascorbigen	Organoheterocyclic compounds	N	C_15_H_15_NO_6_	6.021	304.083	[M − H]−	HMDB0029839	0.766	0.009	2.039
3-(Pyridin-2-yldisulfanyl)propanoic acid	Organoheterocyclic compounds	P	C_8_H_9_NO_2_S_2_	1.241	216.015	[M + H]+	HMDB0247198	0.717	0.003	1.997
Dehydroandrographolide	Organoheterocyclic compounds	N	C_20_H_28_O_4_	6.455	331.191	[M − H]−	HMDB0259665	1.089	0.023	1.928
Cantharidin	Organoheterocyclic compounds	N	C_10_H_12_O_4_	0.145	195.065	[M − H]−	HMDB0249592	0.312	0.020	1.914
Hexadecanoylpyrrolidine	Organoheterocyclic compounds	P	C_20_H_39_NO	10.404	310.310	[M + H]+	HMDB0032740	0.450	0.004	1.885
Neopterin	Organoheterocyclic compounds	N	C_9_H_11_N_5_O_4_	6.192	252.073	[M − H]−	HMDB0000845	1.549	0.026	1.858
Masuda′s compound V	Organoheterocyclic compounds	N	C_12_H_16_N_4_O_7_	4.716	327.095	[M − H]−	HMDB0004256	0.715	0.018	1.846
beta-D-3-Ribofuranosyluric acid	Organoheterocyclic compounds	N	C_10_H_12_N_4_O_7_	4.748	299.063	[M − H]−	HMDB0029920	1.623	0.026	1.771
2-Heptadecylfuran	Organoheterocyclic compounds	P	C_21_H_38_O	10.235	307.300	[M + H]+	HMDB0033608	0.744	0.042	1.503
Diisopropyl sulfide	Organosulfur compounds	P	C_6_H_14_S	1.455	119.090	[M + H]+	HMDB0029579	0.466	0.027	1.582
4-Hydroxycinnamic acid	Phenylpropanoids and polyketides	P	C_9_H_8_O_3_	2.082	147.044	[M + H-H_2_O]+	HMDB0002035	0.585	0.031	1.611

**Notes:** P: positive mode, N: negative mode, VIP: Variable Importance in the Projection.

**Table 3:** The down-regulated metabolites with HMDB ID.

Name	Class	Ion Mode	Formula	RT (min)	*m*/*z*	Adduct	HMDB_ID	log_2_FC	*p*-Value	VIP
N-Acetyldehydroanonaine	Alkaloids and derivatives	P	C_19_H_15_NO_3_	4.854	306.112	[M + H]+	HMDB0041537	−1.451	<0.001	2.224
Moclobemide	Benzenoids	N	C_13_H_17_ClN_2_O_2_	3.006	267.090	[M − H]−	HMDB0015302	−1.273	0.009	2.004
Bisphenol B	Benzenoids	N	C_16_H_18_O_2_	5.584	241.123	[M − H]−	HMDB0245327	−3.162	0.026	1.789
Bakers yeast extract	Benzenoids	N	C_19_H_14_O_2_	5.396	273.091	[M − H]−	HMDB0032173	−0.818	0.031	1.728
Indeno [1,2,3-cd]pyrene	Benzenoids	N	C_22_H_12_	5.395	275.087	[M − H]−	HMDB0253452	−0.729	0.043	1.641
Pentadeca-5,12-dienoylcarnitine	Lipids and lipid-like molecules	P	C_22_H_39_NO_4_	6.921	382.294	[M + H]+	HMDB0241443	−2.270	0.004	1.878
Progesterone	Lipids and lipid-like molecules	P	C_21_H_30_O_2_	7.145	337.214	[M + Na]+	HMDB0001830	−0.905	0.008	1.857
Farnesylcysteine	Lipids and lipid-like molecules	P	C_18_H_31_NO_2_S	7.631	326.214	[M + H]+	HMDB0011627	−0.490	0.019	1.764
Glutarylcarnitine	Lipids and lipid-like molecules	P	C_12_H_22_NO_6_	1.498	276.144	[M + H]+	HMDB0013130	−1.045	0.014	1.753
LysoPG(16:0/0:0)	Lipids and lipid-like molecules	N	C_22_H_45_O_9_P	9.545	483.272	[M − H]−	HMDB0240601	−0.601	0.042	1.679
Falcarinol	Lipids and lipid-like molecules	P	C_17_H_24_O	7.275	245.190	[M + H]+	HMDB0304675	−0.729	0.022	1.642
Farnesol	Lipids and lipid-like molecules	P	C_15_H_26_O	8.196	245.187	[M + Na]+	HMDB0004305	−0.370	0.025	1.633
LysoPC(14:0/0:0)	Lipids and lipid-like molecules	P	C_22_H_46_NO_7_P	8.625	490.290	[M + Na]+	HMDB0010379	−0.378	0.030	1.614
7-Dehydrocholesterol	Lipids and lipid-like molecules	P	C_27_H_44_O	9.428	367.335	[M + H-H_2_O]+	HMDB0000032	−0.579	0.025	1.605
sn-Glycero-3-phosphocholine	Lipids and lipid-like molecules	P	C_8_H_20_NO_6_P	1.327	258.110	[M + H]+	HMDB0000086	−1.955	0.029	1.569
27-Hydroxycholesterol	Lipids and lipid-like molecules	P	C_27_H_46_O_2_	9.433	385.346	[M + H-H_2_O]+	HMDB0002103	−0.580	0.032	1.558
LysoPG(16:0/0:0)	Lipids and lipid-like molecules	N	C_22_H_45_O_9_P	9.545	483.272	[M − H]−	HMDB0240601	−0.601	0.042	1.679
Falcarinol	Lipids and lipid-like molecules	P	C_17_H_24_O	7.275	245.190	[M + H]+	HMDB0304675	−0.729	0.022	1.642
Farnesol	Lipids and lipid-like molecules	P	C_15_H_26_O	8.196	245.187	[M + Na]+	HMDB0004305	−0.370	0.025	1.633
LysoPC(14:0/0:0)	Lipids and lipid-like molecules	P	C_22_H_46_NO_7_P	8.625	490.290	[M + Na]+	HMDB0010379	−0.378	0.030	1.614
7-Dehydrocholesterol	Lipids and lipid-like molecules	P	C_27_H_44_O	9.428	367.335	[M + H-H_2_O]+	HMDB0000032	−0.579	0.025	1.605
sn-Glycero-3-phosphocholine	Lipids and lipid-like molecules	P	C_8_H_20_NO_6_P	1.327	258.110	[M + H]+	HMDB0000086	−1.955	0.029	1.569
27-Hydroxycholesterol	Lipids and lipid-like molecules	P	C_27_H_46_O_2_	9.433	385.346	[M + H-H_2_O]+	HMDB0002103	−0.580	0.032	1.558
cAMP	Nucleosides, nucleotides, and analogues	N	C_10_H_12_N_5_O_6_P	3.723	328.045	[M − H]−	HMDB0000058	−0.663	0.001	2.298
Adenosine	Nucleosides, nucleotides, and analogues	N	C_10_H_13_N_5_O_4_	3.988	266.089	[M − H]−	HMDB0000050	−2.409	0.004	2.240
Riboflavin monophosphate	Nucleosides, nucleotides, and analogues	N	C_17_H_21_N_4_O_9_P	5.237	455.097	[M − H]−	HMDB0001520	−0.472	0.003	2.239
URIDINE 5′-DIPHOSPHOGLUCURONIC ACID	Nucleosides, nucleotides, and analogues	N	C_15_H_22_N_2_O_18_P_2_	1.192	579.028	[M − H]−	HMDB0000935	−1.537	0.002	2.235
Guanosine 5′-diphosphate	Nucleosides, nucleotides, and analogues	N	C_10_H_15_N_5_O_11_P_2_	1.476	442.016	[M − H]−	HMDB0001201	−0.764	0.003	2.143
Adenosine monophosphate	Nucleosides, nucleotides, and analogues	P	C_10_H_14_N_5_O_7_P	5.177	348.071	[M + H]+	HMDB0000045	−1.017	0.003	2.052
ADP-D-ribose	Nucleosides, nucleotides, and analogues	N	C_15_H_23_N_5_O_14_P_2_	1.377	558.064	[M − H]−	HMDB0001178	−0.571	0.007	2.048
ADENOSINE 5′-DIPHOSPHATE	Nucleosides, nucleotides, and analogues	N	C_10_H_15_N_5_O_10_P_2_	1.42	426.022	[M − H]−	HMDB0001341	−0.765	0.009	2.024
Phosphoribosyl formamidocarboxamide	Nucleosides, nucleotides, and analogues	N	C_10_H_15_N_4_O_9_P	1.961	365.050	[M − H]−	HMDB0001439	−1.323	0.010	1.985
Dephospho-CoA	Nucleosides, nucleotides, and analogues	P	C_21_H_35_N_7_O_13_P_2_S	4.911	688.156	[M + H]+	HMDB0001373	−1.340	0.003	1.971
Arabinosylhypoxanthine	Nucleosides, nucleotides, and analogues	P	C_10_H_12_N_4_O_5_	2.125	269.088	[M + H]+	HMDB0003040	−0.723	0.005	1.905
GDP-D-mannose	Nucleosides, nucleotides, and analogues	N	C_16_H_25_N_5_O_16_P_2_	1.847	604.070	[M − H]−	HMDB0001163	−2.361	0.014	1.902
N(6),O(2)-Dimethyladenosine	Nucleosides, nucleotides, and analogues	P	C_12_H_17_N_5_O_4_	5.2	296.136	[M + H]+	HMDB0255287	−0.805	0.004	1.881
5′-Methylthioadenosine	Nucleosides, nucleotides, and analogues	P	C_11_H_15_N_5_O_3_S	5.23	298.097	[M + H]+	HMDB0001173	−0.915	0.007	1.819
5′-Deoxyadenosine	Nucleosides, nucleotides, and analogues	P	C_10_H_13_N_5_O_3_	4.883	252.109	[M + H]+	HMDB0001983	−1.091	0.009	1.795
S-Adenosylhomocysteine	Nucleosides, nucleotides, and analogues	P	C_14_H_20_N_6_O_5_S	2.599	385.128	[M + H]+	HMDB0000939	−0.373	0.009	1.786
S-adenosyl-L-methionine	Nucleosides, nucleotides, and analogues	P	C_15_H_22_N_6_O_5_S	1.37	399.144	[M + H]+	HMDB0001185	−0.761	0.012	1.752
Guanosine	Nucleosides, nucleotides, and analogues	N	C_10_H_13_N_5_O_5_	2.276	282.084	[M − H]−	HMDB0000133	−0.446	0.045	1.745
5′-Phosphoribosyl-N-formylglycinamide	Nucleosides, nucleotides, and analogues	P	C_8_H_15_N_2_O_9_P	1.398	315.058	[M + H]+	HMDB0001308	−0.628	0.015	1.734
UDP-GalNAc	Nucleosides, nucleotides, and analogues	N	C_17_H_27_N_3_O_17_P_2_	1.733	606.075	[M − H]−	HMDB0060522	−0.606	0.043	1.653
DL-O-Phosphoserine	Organic acids and derivatives	N	C_3_H_8_NO_6_P	1.291	184.001	[M − H]−	HMDB0001721	−0.598	0.001	2.252
Asp-Trp	Organic acids and derivatives	P	C_15_H_17_N_3_O_5_	5.273	320.124	[M + H]+	HMDB0028764	−1.159	0.001	2.061
L-Aspartyl-4-phosphate	Organic acids and derivatives	P	C_4_H_8_NO_7_P	1.427	214.011	[M + H]+	HMDB0012250	−0.960	0.003	2.040
5-phosphonooxy-L-lysine	Organic acids and derivatives	N	C_6_H_15_N_2_O_6_P	1.206	241.059	[M − H]−	HMDB0059600	−0.378	0.007	2.020
Hydroxyprolyl-Methionine	Organic acids and derivatives	N	C_10_H_18_N_2_O_4_S	5.049	261.091	[M − H]−	HMDB0028869	−1.147	0.026	1.910
N-Phenylacetylphenylalanine	Organic acids and derivatives	P	C_17_H_17_NO_3_	5.911	284.129	[M + H]+	HMDB0002372	−0.730	0.005	1.889
Nicotianamine	Organic acids and derivatives	N	C_12_H_21_N_3_O_6_	5.164	302.135	[M − H]−	HMDB0255025	−0.560	0.026	1.807
gamma-L-Glutamyl-gamma-L-glutamyl-L-methionine	Organic acids and derivatives	N	C_15_H_25_N_3_O_8_S	5.13	406.129	[M − H]−	HMDB0038674	−0.802	0.031	1.801
4-Guanidinobutanoate	Organic acids and derivatives	P	C_5_H_11_N_3_O_2_	1.412	146.092	[M + H]+	HMDB0003464	−0.711	0.024	1.745
N-Docosahexaenoyl Serine	Organic acids and derivatives	N	C_25_H_37_NO_4_	8.702	414.264	[M − H]−	HMDB0242021	−0.346	0.034	1.705
Prenisteine	Organic acids and derivatives	P	C_8_H_15_NO_2_S	5.505	190.090	[M + H]+	HMDB0012286	−0.369	0.022	1.699
2-n-Propylthiazolidine-4-carboxylic acid	Organic acids and derivatives	P	C_7_H_13_NO_2_S	5.07	176.074	[M + H]+	HMDB0245245	−0.365	0.026	1.670
gamma-Glutamyl-valine	Organic acids and derivatives	N	C_10_H_18_N_2_O_5_	5.086	227.103	[M-H-H_2_O]-	HMDB0011172	−0.446	0.046	1.655
Myristoylglycine	Organic acids and derivatives	N	C_16_H_31_NO_3_	8.376	284.223	[M − H]−	HMDB0013250	−0.527	0.046	1.626
Glycylprolylhydroxyproline	Organic acids and derivatives	P	C_12_H_19_N_3_O_5_	5.205	286.140	[M + H]+	HMDB0002171	−1.103	0.032	1.582
2-Dechloroethylifosfamide	Organic acids and derivatives	P	C_5_H_12_ClN_2_O_2_P	1.441	199.040	[M + H]+	HMDB0013859	−0.442	0.041	1.502
L-Leucyl-L-Valine	Organic acids and derivatives	P	C_11_H_22_N_2_O_3_	5.146	231.170	[M + H]+	HMDB0028942	−0.764	0.038	1.493
Bis((hexahydro-4-methyl-1H-1,4-diazepin-1-yl)thiocarbonyl)disulfide	Organic nitrogen compounds	P	C_14_H_26_N_4_S_4_	4.781	379.111	[M + H]+	HMDB0252285	−0.839	0.018	1.760
5-Hydroxy-N-formylkynurenine	Organic oxygen compounds	P	C_11_H_12_N_2_O_5_	5.257	253.082	[M + H]+	HMDB0004086	−1.823	0.003	1.948
2-Methyl-4′-(methylthio)-2-morpholinopropiophenone	Organic oxygen compounds	P	C_15_H_21_NO_2_S	5.998	280.137	[M + H]+	HMDB0247316	−0.623	0.006	1.836
CMP-2-aminoethylphosphonate	Organic oxygen compounds	N	C_11_H_20_N_4_O_10_P_2_	1.377	429.057	[M − H]−	HMDB0060067	−0.704	0.025	1.779
gamma-Glutamyl-valine	Organic acids and derivatives	N	C_10_H_18_N_2_O_5_	5.086	227.103	[M-H-H_2_O]-	HMDB0011172	−0.446	0.046	1.655
Nicotinamide	Organoheterocyclic compounds	P	C_6_H_6_N_2_O	2.34	123.055	[M + H]+	HMDB0001406	−0.479	<0.001	2.244
Tryptophan 2-C-mannoside	Organoheterocyclic compounds	N	C_17_H_22_N_2_O_7_	4.033	365.135	[M − H]−	HMDB0240296	−0.296	0.003	2.175
Riboflavin cyclic-4′,5′-phosphate	Organoheterocyclic compounds	N	C_17_H_19_N_4_O_8_P	5.208	437.086	[M − H]−	HMDB0059614	−0.428	0.014	2.026
2,4-Dihydroxy-6,7-dimethoxy-2H-1,4-benzoxazin-3(4H)-one	Organoheterocyclic compounds	N	C_10_H_11_NO_6_	5.025	240.051	[M − H]−	HMDB0037548	−1.195	0.009	1.981
Imidazooxazole	Organoheterocyclic compounds	P	C_4_H_3_N_3_O	3.004	110.035	[M + H]+	HMDB0253423	−0.665	0.003	1.974
Metamizol	Organoheterocyclic compounds	N	C_13_H_17_N_3_O_4_S	5.024	310.086	[M − H]−	HMDB0254502	−1.089	0.017	1.949
delta9-Tetrahydrocannabinol	Organoheterocyclic compounds	P	C_21_H_30_O_2_	7.145	315.231	[M + H]+	HMDB0014613	−0.885	0.004	1.939
Xanthine	Organoheterocyclic compounds	N	C_5_H_4_N_4_O_2_	1.633	151.025	[M − H]−	HMDB0000292	−0.652	0.016	1.933
4-Amino-5-hydroxymethyl-2-methylpyrimidine	Organoheterocyclic compounds	P	C_6_H_9_N_3_O	1.37	122.071	[M + H-H_2_O]+	HMDB0247327	−0.514	0.011	1.812
3-Methyladenine	Organoheterocyclic compounds	P	C_6_H_7_N_5_	1.839	150.077	[M + H]+	HMDB0011600	−0.283	0.010	1.778
2-Amino-1,6-dimethylfuro [3,2-e]imidazo [4,5-b]pyridine	Organoheterocyclic compounds	P	C_10_H_10_N_4_O	5.492	203.093	[M + H]+	HMDB0035175	−0.605	0.013	1.740
guanine	Organoheterocyclic compounds	P	C_5_H_5_N_5_O	3.155	152.057	[M + H]+	HMDB0000132	−0.842	0.039	1.702
Quinaldic acid	Organoheterocyclic compounds	P	C_10_H_7_NO_2_	1.227	196.037	[M + Na]+	HMDB0000842	−0.326	0.019	1.643
3,N(4)-Ethenodeoxycytidine	Organoheterocyclic compounds	P	C_11_H_13_N_3_O_4_	4.942	252.098	[M + H]+	HMDB0246084	−0.691	0.034	1.603
Thiamine	Organoheterocyclic compounds	P	C_12_H_16_N_4_OS	1.384	265.111	[M + H]+	HMDB0000235	−0.529	0.046	1.522
6-Hydroxymelatonin	Organoheterocyclic compounds	P	C_13_H_16_N_2_O_3_	1.27	271.105	[M + Na]+	HMDB0004081	−0.395	0.036	1.514

**Notes:** P: positive mode, N: negative mode, VIP: Variable Importance in the Projection. To further explore the potential metabolic mechanism, we performed clustering, KEGG classification, and exploratory pathway analysis based on the extracted 138 metabolites to identify the physiological pathways. As shown in Figure 6A, the metabolites were divided into 10 classes, and there were significant differences in each class between the Sham and MCAO groups, especially in the lipid and lipid-like molecules and nucleosides, nucleotides, and analogues. KEGG analysis showed that these metabolites had potential correlations with amino acid metabolism, nucleoside metabolism, and lipid metabolism (Figure 6B), and the pathways may be related to purine metabolism and amino acid metabolism, such as the Pantothenate and CoA biosynthesis, Arginine biosynthesis, Arginine and proline metabolism, and the Histidine metabolism (Figure 6C).

**Figure 6:**
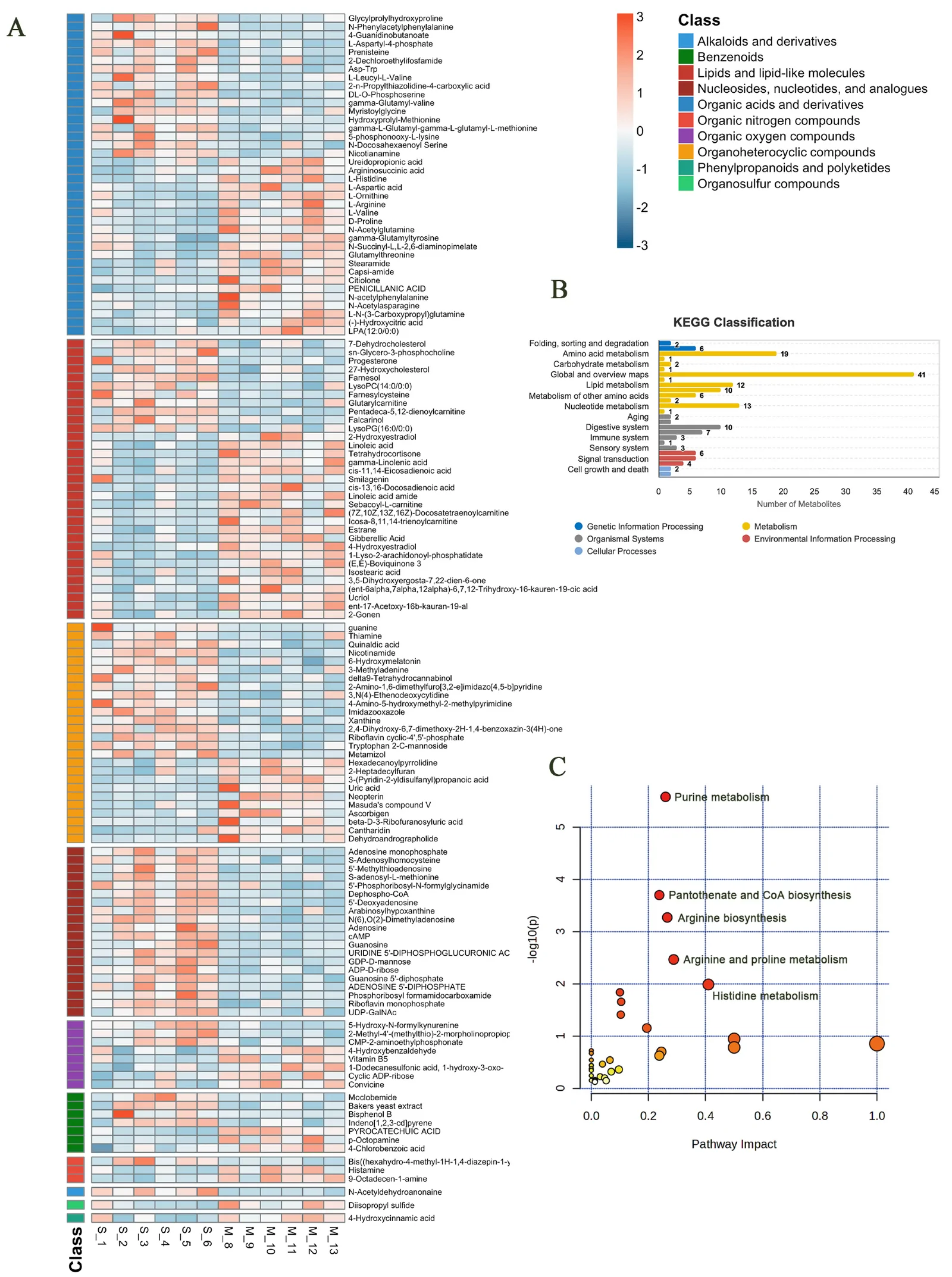
Signature of identified metabolites with HMDB ID. (**A**) Heatmap of 138 identified metabolites that were differentially expressed in the two groups (*p* < 0.05). The colours changing from red to blue indicates the expression level variations in metabolites, the red plots indicate the up-regulated metabolites, and the blue plots indicate the down-regulated metabolites. (**B**) KEGG classification of the 138 identified metabolites. (**C**) Potential pathways analysis of 138 identified metabolites. The horizontal axis represents pathway impact, which is the extent of differential metabolites’ impact on pathways, and the larger the pathway impact, the bigger the dot size. The vertical axis represents the enrichment-level *p*-value, and the smaller the *p*-value, the more the differential metabolites are annotated to the pathway.

To more clearly present the variations in specific metabolites, violin plots were generated for a set of core representative metabolites, displaying their distribution profiles and changes between the Sham and MCAO groups (Supplementary Figures S1 and S2).

## 4. Discussion

Ischemic stroke (IS), a major contributor to global mortality and disability, is precipitated by vascular occlusion that disrupts cerebral blood flow, thereby inducing local ischemia, hypoxia, and subsequent neurological injury [32]. The MCAO model is widely employed because it closely recapitulates the pathophysiological features of human ischemic stroke [33]. In this study, we successfully generated the MCAO model in C57BL/6J mice, which was verified through neurological deficit scores and quantification of infarct volume. The onset of IS triggers profound shifts in metabolite levels and a significant dysregulation of metabolic pathways. It has been reported that IS involves a complex interplay among oxidative stress, regulated cell death, and lipid metabolism [34,35]. Moreover, several therapies have been discovered that treat IS by targeting these metabolic processes [36,37]. Therefore, investigating the metabolic mechanism underlying IS is necessary and important. Metabolomics is a scientific discipline that provides high-throughput characterization and precise profiling of metabolites in cells, organs, tissues, or biofluids using advanced analytical techniques [38], and it plays a major role in clinical practice, as the quantification of metabolites constitutes over 95% of the workload in clinical laboratories worldwide. We employed untargeted metabolomics to investigate the metabolic alterations in the ischemic penumbra of MCAO mice in the current study.

Analysis of the overall metabolite profile revealed that “lipids and lipid-like molecules”, “organic acids and derivatives”, “organohererocyclic compounds”, “benzenoids”, “organic oxygen compounds”, and “nucleosides, nucleotides, and analogues” classes were the six most predominant among the 1836 metabolites identified. Exploratory KEGG pathway annotation of metabolites selected based on nominal statistical significance suggested potential enrichment in “amino acid metabolism”, “carbohydrate metabolism”, “lipid metabolism”, “metabolism of cofactors and vitamins”, and “nucleotide metabolism”. Moreover, among metabolites possessing HMDB numbers, large proportions were categorized as “lipids and lipid-like molecules”, “organic acids and derivatives”, “organic oxygen compounds”, “organoheterocyclic compounds”, and “nucleosides, nucleotides, and analogues”. Taken together, these data indicated that IS may be associated with alterations in lipid metabolism, nucleotide metabolism, and amino acid metabolism.

### 4.1. Lipid Metabolism in Ischemic Stroke

The MCAO group exhibited heterogeneous differences in lipid metabolites, with some increased and others decreased levels, suggesting that lipid metabolism is substantially disturbed at 72 h after ischemic brain injury.

One notable finding was the putatively annotated steroid-related metabolite estrane (*p* = 0.001, VIP = 2.058). Estrane is the parent structure of many estrogenic compounds, and its increase may indicate altered steroid-associated metabolism after MCAO. Estrogens have been reported to participate in multiple biological processes relevant to ischemic brain injury [39], while 17β-estradiol (E2) contributes to central nervous system (CNS) homeostasis and injury resilience [40]. In the present study, 2-Hydroxyestradiol and 4-Hydroxyestradiol were also increased after MCAO (2-Hydroxyestradiol, *p* = 0.015, VIP = 1.7, 4-Hydroxyestradiol, *p* = 0.017, VIP = 1.872), which is directionally consistent with the increase in estrane. These are important metabolites of E2 and promote brain development and protection [41]. Our study also corroborates recent metabolic profiling findings in IS patient serum [42]. However, given the structural similarity of steroid metabolites and the limitations of untargeted LC-MS annotation without confirmation by authentic standards, these metabolites should be regarded as putatively annotated compounds. Therefore, their biological significance requires targeted LC-MS/MS validation and functional investigation.

We also detected an increase in linoleic acid (LA, *p* = 0.002, VIP = 1.972), an essential fatty acid that cannot be synthesized de novo. Previous studies have suggested that LA-derived metabolites may participate in the regulation of neuronal signalling after ischemic injury [43]. Clinical studies also reported associations between higher LA intake or tissue content and lower risk of IS [44,45]. In our dataset, linoleic acid amide (LAA, *p* = 0.002, VIP = 1.986) and gamma-linolenic acid (GLA, *p* = 0.003, VIP = 1.942) were also increased, showing a similar trend to LA. These findings may reflect remodelling of LA-related lipid metabolism after ischemic injury. However, LA metabolism can generate both potentially protective and harmful lipid mediators. For example, oxidized LA products such as 13-HPODE have been associated with inflammatory responses [46,47,48]. Therefore, the observed increase in LA-related metabolites here should be interpreted as evidence of altered LA metabolism rather than a protective response. Further targeted and functional studies are needed to determine whether these changes contribute to injury progression and compensatory adaptation.

Among the down-regulated lipid-related metabolites, Progesterone (Prog) showed a significant decrease after MCAO (*p* = 0.008, VIP = 1.857). Progesterone is involved in steroid hormone synthesis and has biological functions in the central nervous and immune systems [49]. Previous experimental research has shown that Prog serves as a candidate neuroprotective factor and holds promising therapeutic potential for IS [50,51], which is related to inflammation, neurotoxicity, mitochondrial function, edema, apoptosis, and neurogenesis [52,53,54]. However, the interpretation of progesterone-related findings remains complex. Evidence regarding its therapeutic effects in ischemic stroke is not fully consistent, and clinical evidence supporting its efficacy remains limited. In addition, whether progesterone promotes neurogenesis remains controversial [55,56,57]. Therefore, the decrease in progesterone observed in our study should be interpreted cautiously as a metabolic alteration associated with MCAO rather than direct evidence of impaired progesterone-mediated neuroprotection.

Overall, our exploratory findings suggest that MCAO may be associated with broad disturbances in lipid metabolism, including changes in steroid-related metabolites, LA-related metabolites, and progesterone. These alterations may provide clues to lipid metabolic remodelling during ischemic injury, but their mechanistic roles require further validation. Nevertheless, these steroid-related metabolites were detected in male mice and should be interpreted within the context of the present male MCAO model.

### 4.2. Nucleotide Metabolism in Ischemic Stroke

Among the metabolites showing nominal changes after MCAO, several nucleotide-related metabolites exhibited an overall downward trend, reflecting possible disruption of nucleotide turnover and cellular energy-related metabolism in ischemic brain injury.

A representative downregulated metabolite in this pathway is cyclic adenosine monophosphate (cAMP, *p* = 0.001, VIP = 2.298), a second messenger involved in multiple CNS signalling processes including neuroplasticity, neurotransmission, neuronal survival, myelination, and neuroinflammatory regulation [58]. Previous studies have suggested that cAMP-related signalling may modulate immune-mediated injury by affecting neuronal, astrocytic, endothelial, and microglial functions [59]. In the present study, the reduction in cAMP may reflect altered second messenger signalling and energy-related metabolic stress after ischemic injury. However, because cAMP signalling activity was not directly measured, this interpretation should be considered inferential.

In addition to cAMP, adenosine (Ado)-related metabolites also showed a prominent overall reduction including Adenosine monophosphate (*p* = 0.003, VIP = 2.052) and Adenosine 5′-diphosphate (*p* = 0.009, VIP = 2.024). As a key ATP-derived metabolite, Ado exerts neuroprotection primarily via A1 receptor activation by inhibiting glutamatergic excitotoxicity and anti-inflammatory effects via A2A receptor activation on infiltrating immune cells during the subacute phase of IS [60,61]. Under acute ischemic conditions, rapid ATP consumption typically elevates Ado levels [62]. In contrast, our metabolomic analysis was performed three days after MCAO and showed an overall decrease in nucleotide-related metabolites. This discrepancy may reflect the temporal specificity of nucleotide metabolism after ischemic injury. Previous studies have also shown that energy-related metabolites can vary across brain regions and time points after ischemia, with early metabolic decline and later region-specific changes in energy reserves [63]. The decrease in nucleotide-related metabolites may therefore be associated with sustained energy metabolic disturbance, mitochondrial stress, or reduced nucleotide availability during the subacute post-ischemic phase. Mitochondrial dysfunction and energy failure are central features of acute ischemic injury and may also contribute to persistent tissue damage and delayed neuronal vulnerability [64]. However, the current study did not directly assess mitochondrial function, ATP turnover, or enzymatic activity. Therefore, the observed nucleotide changes should be interpreted as metabolomic evidence suggestive of altered energy-related metabolism rather than direct proof of mitochondrial dysfunction.

Guanosine (Guo) is a nucleotide metabolite defined by its neurotrophic and regenerative capacities, through which it acts as a potent neuromodulator in neurological disorders [65,66]. It has been demonstrated that Guo exerts potential beneficial effects of Guo administration in MCAO models [67]. Our findings at 3 days after MCAO differed from some results obtained through rapid or early-stage monitoring [68]. Similar temporal differences were also observed for adenosine-related metabolites. These discrepancies further support the concept that nucleotide metabolism after cerebral ischemia is highly dynamic and strongly dependent on sampling time.

In summary, the observed downregulation of nucleotide metabolism, including cAMP, Ado, and Guo, suggests possible disruption of nucleotide and energy-related metabolism at 72 h after MCAO. Our findings may highlight the temporal specificity of nucleotide metabolic changes after ischemic injury while further time-course studies combining targeted metabolomics are needed to find out their dynamic roles of ATP metabolism in brain ischemia progression.

### 4.3. Amino Acid Metabolism in Ischemic Stroke

As shown in the KEGG map, amino acid metabolism could be another affected pathway after MCAO. Amino acids and their metabolites are essential to neurological health, acting not only as building blocks for proteins but also as key neurotransmitters and signalling molecules [69].

In the present study, both Histidine (His, *p* = 0.023, VIP = 1.660) and Arginine (Arg, *p* = 0.041, VIP = 1.555) were upregulated after MCAO, indicating active alterations in amino acid metabolism during ischemic brain injury. Histidine is an essential amino acid with critical roles in maintaining tissue homeostasis and neural function [70]. Emerging evidence indicates that activation of the histaminergic system confers long-term neuroprotection against cerebral ischemic injury, particularly through modulating astrocyte function [71]. Histidine serves as the direct precursor for histamine synthesis via histidine decarboxylase, and histamine further regulates neuroimmune responses, oxidative stress, and inflammatory reactions [72]. Exogenous histidine has been shown to exert neuroprotection by increasing histamine production as well [73]. The significant upregulation of histidine observed in our study may represent an endogenous adaptive response aimed at enhancing histamine-mediated neuroprotection and counteracting ischemic damage. However, because histamine levels, histidine decarboxylase activity, and downstream histaminergic signalling were not measured, this finding should not be interpreted as direct evidence of histamine-mediated neuroprotection.

Arginine, a nitrogen-rich amino acid, functions as a pivotal precursor for nitric oxide (NO) synthesis and is essential for maintaining endothelial function, cerebral blood flow, and mitochondrial integrity [74,75]. Arg is widely recognized as a protective metabolite in ischemic stroke by inhibiting inflammatory responses through suppressing HIF-1α and LDHA [76]. It also improves cerebral microcirculation during the prothrombotic state induced by ischemic injury [77]. The marked upregulation of Arg in our results could be further supports its role in endogenous defence mechanisms against brain ischemia, likely by enhancing NO-dependent vascular regulation and anti-inflammatory pathways.

Taken together, the nominally upregulation of His and Arg suggests that amino acid metabolism may be associated with the metabolic response to ischemic injury, which may contribute to neuroprotection, anti-inflammation, and the maintenance of vascular function, emphasizing the importance of amino acid metabolism in the pathophysiology of ischemic stroke. However, the current untargeted metabolomics data do not establish causality. Future studies integrating targeted amino acid quantification, enzyme activity assays, inflammatory markers, and functional validation are needed to determine the mechanistic roles of these amino acid alterations in ischemic stroke.

## 5. Conclusions

In this discovery-stage untargeted metabolomics study, we profiled metabolic differences in the cerebral cortex of mice 72 h after MCAO. The observed metabolites included lipids and lipid-like molecules, organic acids and derivatives, organic oxygen compounds, organoheterocyclic compounds, and nucleosides, nucleotides, and analogues. Exploratory pathway analysis suggested possible associations with lipid, nucleotide, and amino acid metabolism. However, these metabolites and enriched pathways should be regarded as hypothesis-generating observations. Further validation study of metabolites (including estrane, LA, Prog, Ado, Guo, His, and Arg) and related pathophysiological processes will contribute to the mechanism of ischemic stroke and clinical therapies.

There are three limitations to this study. Firstly, by concentrating solely on the 3-day time point, we could not track the transient metabolic dynamics of cerebral ischemia, and the outcome of this discovery-stage only reflects the 72 h phase after MCAO. Secondly, the reliance on the MCAO model alone constrains the direct clinical applicability of our conclusions. We thus advocate for future investigations that integrate longitudinal designs and human samples to better inform clinical translation. In addition, untargeted metabolomics is semi-quantitative, and this study did not use FDR as a filtering criterion, which may limit the accuracy of biomarker identification. Future studies with larger sample sizes, independent validation cohorts, targeted LC-MS/MS quantification, and appropriate multiple-testing correction are required to confirm the robustness of these findings.

## Figures and Tables

**Table 1:** Gradient elution program.

Time (min)	A%	B%
0	98	2
1.5	98	2
3	15	85
10	0	100
10.1	98	2
11	98	2
12	98	2
12	98	2

## Data Availability

The data presented in this study are available on request from the corresponding author due to privacy and further study.

## Supplementary Materials

The following supporting information can be downloaded at: https://www.mdpi.com/article/10.3390/metabo16080544/s1, Table S1: The up-regulated metabolites with FDR; Table S2: The down-regulated metabolites with FDR; Figure S1: Violin plot of upregulated metabolites with HMDB ID; Figure S2: Violin plot of downregulated metabolites with HMDB ID.
